# Effects of kinesiology taping timing on recovery in the lower limbs

**DOI:** 10.3389/fphys.2025.1588339

**Published:** 2025-04-28

**Authors:** Chia-Yu Tang, Ying-Che Huang, Fu-Shun Hsu, Chia-Hsien Yu, Chang-Chi Lai, Szu-Kai Fu

**Affiliations:** ^1^ Graduate Institute of Sports Training, College of Kinesiology, University of Taipei, Taipei, Taiwan; ^2^ Department of Anesthesia and Critical Care Medicine, Yangming Branch, Taipei City Hospital, Taipei, Taiwan; ^3^ Department of Exercise and Health Sciences, College of Kinesiology, University of Taipei, Taipei, Taiwan; ^4^ Department of Urology, Yangming Branch, Taipei City Hospital, Taipei, Taiwan; ^5^ Department of Urology, College of Medicine, National Taiwan University, Taipei, Taiwan

**Keywords:** EIMD, neuromuscular efficiency, kinesiology taping, neuromuscular recovery strategy, DOMS, sensorimotor feedback, peripheral fatigue

## Abstract

**Background:**

Kinesiology taping (KT) is widely used to support muscle function and recovery, but its optimal application timing remains unclear. While some suggest pre-exercise KT provides protective benefits, others propose post-exercise KT aids recovery. Eccentric contractions often lead to eccentric exercise-induced muscle damage (EIMD), causing strength loss, soreness, and reduced range of motion. Whether KT timing influences its effectiveness in mitigating or accelerating EIMD recovery requires further investigation.

**Purpose:**

This study examined whether KT, applied before (KT-pre) or after (KT-post) eccentric exercise of the knee extensors, could mitigate or hasten recovery from EIMD in the lower limbs.

**Methods:**

12 healthy adult males (22.0 ± 1.7 years) participated in a repeated-measures crossover study under three conditions: KT-pre, KT-post, and a no-taping control (CON). Participants performed 72 eccentric contractions of the knee extensors on the non-dominant leg using an isokinetic dynamometer. Outcome measures included maximal voluntary isometric contraction (MVIC) normalized to body weight, rate of force development (RFD) in the 0–200 ms interval, neuromuscular efficiency (NME, defined as the ratio of peak torque to integrated electromyography), active ROM of knee flexion (measured via goniometry), and subjective muscle soreness (100-mm visual analogue scale). Assessments were conducted at baseline and at 0-, 24-, and 48-h post-exercise.

**Results:**

When expressed as a percentage of baseline, both peak torque and RFD in the 0–200 ms interval declined significantly at 0- and 24-h post-exercise (*p* < 0.05) in all groups, with no significant intergroup differences. The iEMG parameter remained unchanged. NME declined significantly at 0 h (*p* < 0.05) in all conditions; however, at 24 h, the KT-pre group exhibited significantly higher NME than the control (79.3% ± 12.8% vs. 94.4% ± 17.4%, *p* = 0.0052). Active ROM decreased and subjective muscle soreness increased significantly at 0 and 24 h (*p* < 0.05) across all groups, with no significant intergroup differences.

**Conclusion:**

Although KT-pre demonstrated a short-term protective effect immediately after eccentric exercise, neither pre- nor post-exercise taping significantly mitigated muscle damage or enhanced recovery. Further research is needed to clarify KT’s long-term benefits and its effects on EIMD in other muscle groups.

## 1 Introduction

KT has gained considerable attention in clinical and athletic settings due to its convenience, affordability, and noninvasive nature (Nelson, 2016). It is purported to improve circulation, support soft tissues, and provide enhanced proprioceptive feedback, potentially reducing pain and stabilizing joints during or after exercise (Kim et al., 2016). While some studies report that KT can augment neuromuscular recruitment and help sustain force output under fatigue (Magalhães et al., 2016), others have found inconsistent or negligible effects on EIMD recovery (Ozmen et al., 2016). Recent systematic reviews have further explored KT’s broader applications in both healthy and clinical populations. For example, its use has been shown to modulate sensorimotor feedback, balance, and pain modulation in a range of conditions, including musculoskeletal injuries and chronic diseases (Biz et al., 2022; Lin et al., 2021). These findings support KT’s potential as a versatile tool not only for performance enhancement but also for rehabilitation. These conflicting findings highlight the need for systematic research examining the timing of KT application—particularly its prophylactic (KT-pre) versus rehabilitative (KT-post) use—during eccentric loading of the knee extensors.

EIMD commonly arises from unaccustomed or high-intensity exercise, particularly when eccentric contractions are involved (Chen and Hsieh, 2001) This damage is frequently observed in the lower limbs, where activities such as downhill running, plyometrics, and eccentric squats can induce microtears in muscle fibers and trigger inflammatory responses (Chen et al., 2013; Hicks et al., 2016; Hyldahl and Hubal, 2014). Symptoms of EIMD include force reduction, delayed-onset muscle soreness (DOMS), and decreased range of motion, all of which can disrupt training regimens for both athletes and recreational exercisers (Chen et al., 2007). Although these symptoms often resolve within several days, the functional deficits and potential for reinjury underscore the importance of identifying effective preventive and rehabilitative strategies (Chen et al., 2019).

Numerous approaches have been proposed to mitigate EIMD or expedite its recovery. Nutritional interventions—such as protein supplementation, antioxidant consumption, or creatine loading—have shown promise in certain populations (Cooke et al., 2009; Howatson and van Someren, 2008). Additionally, the repeated bout effect (RBE) suggests that a second eccentric exercise session induces less damage than the initial session, likely through adaptations to the muscle’s structural and neural components (Chen et al., 2013). Beyond these, various recovery techniques—including massage, cold-water immersion, and compression garments—are frequently used to reduce inflammation and speed up tissue repair (Dupuy et al., 2018; Nédélec et al., 2013). However, logistical barriers and individual variability often limit the consistent application or efficacy of these methods.

Assessing the effectiveness of KT in the context of EIMD involves monitoring neuromuscular efficiency (NME) and RFD. Reductions in NME can reflect an increased neuromuscular effort required to achieve the same force, often attributable to both central and peripheral fatigue (Aagaard et al., 2002; Maffiuletti et al., 2016). Meanwhile, a decrease in RFD—especially in the early phase (0–200 ms)—is indicative of compromised explosive strength, a key concern following eccentric damage (Vila-Chã et al., 2012). If KT provides structural or proprioceptive support, it may help maintain NME and attenuate RFD losses after intense eccentric bouts.

Given the prevalence of lower-limb EIMD and the widespread use of KT as both a preventive and rehabilitative tool, there is a compelling rationale to investigate KT-pre versus KT-post application within a controlled crossover framework. Building upon prior evidence, the present study aimed to determine whether KT, when applied before or after eccentric exercise of the knee extensors, could reduce indices of EIMD or accelerate recovery. We hypothesized that KT-pre would offer immediate protective benefits against eccentric-induced muscle damage, whereas KT-post could facilitate faster return of strength and function. By integrating measures of NME and RFD, this research sought to elucidate the specific contributions of KT to neuromuscular function in the presence of EIMD.

## 2 Materials and methods

### 2.1 Study design

**FIGURE 1:**
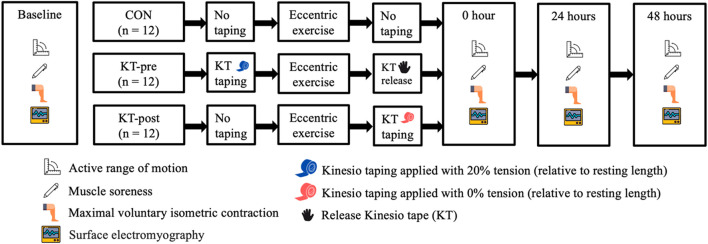
Study design.

This study employed a repeated-measures crossover design involving three interventions: (1) KT-pre, with kinesiology tape applied before eccentric exercise; (2) KT-post, with tape applied immediately after eccentric exercise; and (3) a control condition (CON) without tape application. Participants were randomly assigned to one of six possible intervention sequences (3! = 6), with a one-month washout period between each. Evaluations were conducted at baseline and at 0-, 24-, and 48-h post-exercise, thereby encompassing both immediate and short-term recovery outcomes (Figure 1).

### 2.2 Participants

Twelve healthy adult males (age: 22.0 ± 1.7 years; height: 173.7 ± 7.0 cm; weight: 70.0 ± 11.6 kg) volunteered for this study. Inclusion criteria required no lower-limb musculoskeletal injuries within the preceding 6 months and the ability to safely perform maximal knee extensor contractions. Prior to participating, everyone received detailed information regarding all procedures, potential risks, and benefits, and provided written informed consent. The study protocol was approved by the Institutional Review Board of the University of Taipei (Approval No. 2024-021, Taipei, Taiwan) and adhered to the principles outlined in the Declaration of Helsinki.

### 2.3 Procedures

All participants completed a familiarization session on the isokinetic dynamometer to learn the eccentric exercise protocol. The following outcome measures were recorded: active ROM via goniometry, subjective muscle soreness on a 100-mm visual analogue scale, MVIC of the knee extensors at 30° of flexion, neuromuscular efficiency (NME) derived from surface electromyographic (sEMG) signals during MVIC, and RFD in the 0–200 ms interval. Each intervention was tested at baseline and reassessed at 0-, 24-, and 48-h post-exercise.

#### 2.3.1 Eccentric exercise

Eccentric loading of the quadriceps was performed by the BIODEX System 4 dynamometer (Biodex Medical Systems, Shirley, NY, United States) (Chen and Hsieh, 2001). Each participant began with 10 submaximal warm-up contractions (5 knee flexions and 5 knee extensions) at 60°/s. The primary protocol required 6 sets of 12 maximal eccentric knee extensions at 30°/s, moving from 20° to 90° of knee flexion (Hicks et al., 2016). Upon reaching 90°, participants ceased exertion, and the lever arm passively returned to 20°, initiating the next repetition. A 2-min rest interval was provided between sets. Throughout the procedure, participants were seated with the trunk stabilized, and visual plus verbal feedback was given to encourage maximal effort.

#### 2.3.2 Kinesiology taping

**FIGURE 2:**
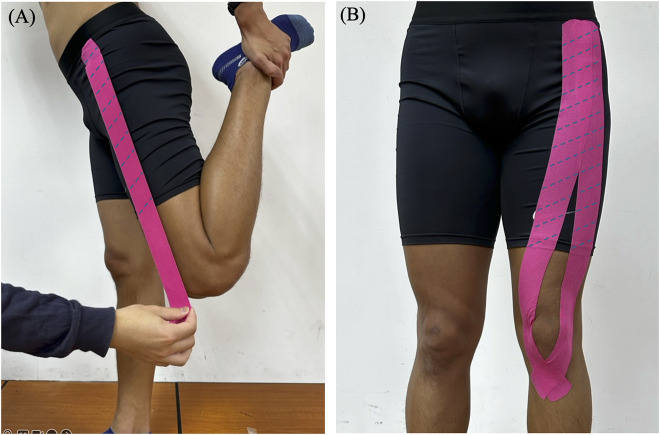
Application of Kinesio tape to the rectus femoris muscle. **(A)** Stretched position **(B)** Taping configuration.

The taping procedure was administered by a certified athletic trainer with over 1 year of experience. First, the distance from the rectus femoris origin (anterior inferior iliac spine) to its insertion (tibial tuberosity) was measured (Vo et al., 2023). NITTO Medical Kinesiology Tape (NK-50, Japan) was cut to 1.25 times this measured length, providing a 20% stretch upon application. In the KT-pre condition, participants stood upright, flexing the knee to maintain the rectus femoris in a stretched position while using the ipsilateral arm to hold the ankle (and the free arm to maintain balance) (Figure 2a). Two I-shaped strips were applied from the AIIS to the tibial tuberosity, circumventing the patella, to cover the rectus femoris (Figure 2b). In the KT-post condition, the same taping procedure was performed immediately upon completion of the eccentric exercise protocol, and no taping was applied in the Control condition.

#### 2.3.3 Range of motion

Active knee joint range of motion (ROM) was measured with a goniometer. Markers were placed on three anatomical reference points (the lateral midpoints of the femur and fibula, and the lateral aspect of the knee). Participants stood on a 10-cm step with their non-dominant foot extended off the step, maintaining a hand on the wall for balance and the other hand on the hip. Active knee flexion ROM of the non-dominant leg was then recorded.

#### 2.3.4 Perceived muscle soreness

Perceived muscle soreness of the knee extensors was assessed using a 100-mm visual analogue scale (VAS), where 0 mm indicates no pain and 100 mm represents extreme pain (Burnett et al., 2010). Participants assumed a standing posture with hands on hips and performed one squat, descending from 0° to 90° of knee flexion and then returning to full extension. During this motion, they were asked to rate their perceived muscle soreness of the knee extensors on the VAS.

#### 2.3.5 Maximal voluntary isometric contraction

MVIC of the knee extensors was measured using a Biodex System 4 dynamometer. Participants sat with hips flexed at 85° and knees flexed at 30°, aligning the lateral femoral condyle with the dynamometer’s axis of rotation. Straps were used to secure the trunk, waist, and distal thigh to minimize extraneous movement. Participants performed three maximal isometric contractions, each lasting 3 s and separated by 60-s rest intervals. The highest peak torque (in N·m) recorded from these three trials was used for analysis (Fu et al., 2021). Rate of force development (RFD) was calculated from the slope of the torque–time curve during MVIC trials (Aagaard et al., 2002). Specifically, RFD was determined in the 0–200 ms window (ΔTorque/ΔTime), expressed in N·m/s (Maffiuletti et al., 2016). Participants were instructed not to pre-load force prior to the test trigger, and to exert maximal effort as quickly as possible. The average of three valid MVIC trials was taken as the representative RFD value.

#### 2.3.6 Neuromuscular efficiency

NME of the knee extensors was assessed by simultaneously recording peak torque and surface electromyography (EMG) signals during MVIC using the myoMUSCLE EMG system (myoRESEARCH 3.14, Noraxon, Scottsdale, AZ, United States). Rectus femoris EMG sensors (Model 548 DTS Lossless EMG sensor, Noraxon) were placed at the midpoint between the anterior inferior iliac spine (AIIS) and the superior border of the patella, with an inter-electrode distance of 20 mm (Hermens et al., 2000). The sampling rate was set to 1,000 Hz, and the EMG signal was full-wave rectified and smoothed using a 100 Hz low-pass filter. The integrated EMG (iEMG) for each MVIC trial was computed over a 1-s plateau. NME was then calculated as peak torque (N·m) divided by iEMG (mV·s), reflecting the torque generated per unit of EMG activity (David et al., 2008).

### 2.4 Statistical analysis

Data were analyzed using GraphPad Prism 10.2 (GraphPad Software, Inc., San Diego, CA, United States). All dependent variables are reported as mean ± standard deviation (SD), with the significance level set at α = 0.05. The Shapiro–Wilk test confirmed normality, while Levene’s test and Mauchly’s test assessed homogeneity of variances and sphericity, respectively. A two-way repeated-measures ANOVA (Intervention × Time) was then conducted for each outcome measure. If interaction effects were significant, Sidak multiple comparisons determined whether post-exercise values (0, 24, 48 h) differed from baseline within each intervention. Additionally, Tukey multiple comparisons assessed differences among the three interventions at each time point. To account for individual variability, performance metrics were normalized to baseline by computing (post-test ÷ pre-test) × 100%, reported as mean ± SD in percentages (%) and rounded to the nearest whole number.

## 3 Results

### 3.1 Test of homogeneity

All data are presented as mean ± standard deviation (SD). Baseline comparisons using repeated-measures ANOVA revealed no significant differences among the CON, KT-post, and KT-pre groups for knee extensor performance and muscle damage indicators, confirming the homogeneity of baseline values (Table 1).

**TABLE 1:** Test of homogeneity.

Outcome Measures	CON (n = 12)	KT-post (n = 12)	KT-pre (n = 12)	*p*-Value
MVIC of Knee Extensors				
Peak Torque (Nm/kg)	1.57 ± 0.28	1.52 ± 0.20	1.46 ± 0.14	0.741
RFD (0–200 ms)	5.45 ± 1.48	5.32 ± 1.37	4.99 ± 0.75	0.921
Neuromuscular Efficiency	0.39 ± 0.15	0.36 ± 0.17	0.31 ± 0.15	0.689
Muscle Damage				
Active ROM of Knee Flexion	90.06 ± 7.75	81.50 ± 6.50	83.13 ± 3.78	0.179
Perceived Muscle Soreness Scale	10.71 ± 4.77	10.66 ± 2.63	9.84 ± 3.76	0.780

Note: CON: No taping; KT-post: Taping after eccentric exercise; KT-pre: Taping during eccentric exercise. Maximal voluntary isometric contraction (MVIC), range of motion (ROM). Significant difference between groups (p < 0.05).

### 3.2 Maximal voluntary isometric contraction of knee extensors

**FIGURE 3:**
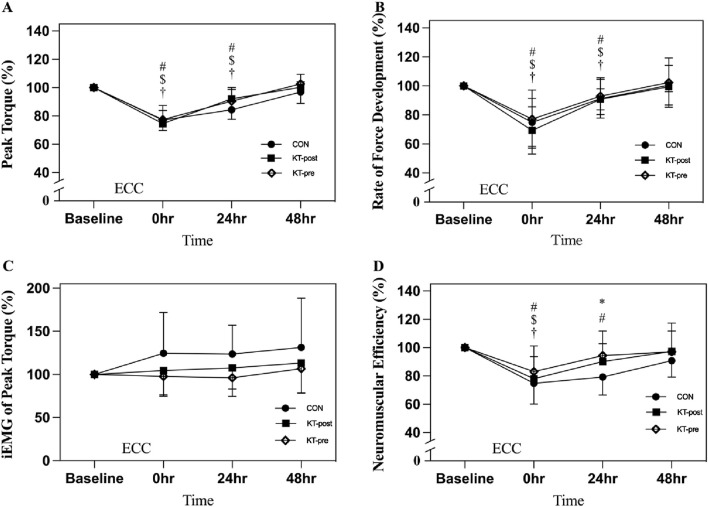
Comparison of peak torque during maximum voluntary isometric contractions of the knee extensors. Note: Data are expressed as mean (%). No taping (CON); taping after eccentric exercise (KT-post); taping during eccentric exercise (KT-pre). **(A)** Peak torque; **(B)** Rate of force development; **(C)** iEMG of peak torque; **(D)** Neuromuscular Efficiency. # represents that the CON group is significantly lower than the baseline after intervention. $ represents that the KT-post group is significantly lower than baseline after intervention. † represents that the KT-pre group is significantly lower than baseline after intervention. * indicates a significant difference between CON and KT-pre at the same time point. *p* < 0.05 indicates a statistically significant difference within and between groups.

MVIC values of the knee extensors, as measured by an isokinetic dynamometer, are presented in Figure 3. When expressed as a percentage of baseline [(post-test/pre-test) × 100%], both peak torque and the RFD in the 0–200 ms interval exhibited statistically significant declines at 0- and 24-h post-exercise (*p* < 0.05) across all groups; however, no significant differences were observed among the groups at any time point (Figures 3A,B). No significant changes were observed in the iEMG parameter (Figure 3C). However, neuromuscular efficiency, defined as the ratio of peak torque to iEMG, exhibited statistically significant declines at 0 h post-exercise (*p* < 0.05) across all groups. Moreover, at 24 h post-exercise, a significant difference was observed between the CON and KT-pre groups (79.3% ± 12.8% vs. 94.4% ± 17.4%, *p* = 0.0052) (Figure 3D).

### 3.3 Muscle damage indicators

**FIGURE 4:**
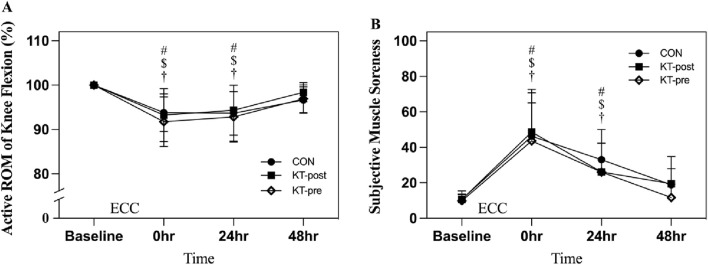
Comparison of muscle damage of the knee extensors. Note: Data are expressed as mean (%). No taping (CON); taping after eccentric exercise (KT-post); taping during eccentric exercise (KT-pre). **(A)** Active ROM of knee flexion; **(B)** Subjective muscle soreness. # represents that the CON group is significantly lower than the baseline after intervention. $ represents that the KT-post group is significantly lower than baseline after intervention. † represents that the KT-pre group is significantly lower than baseline after intervention.

Active ROM of knee flexion measured via goniometry demonstrated a significant decrease at 0- and 24-h post-exercise (*p* < 0.05) across all groups; however, no significant differences were observed among the groups at any time point (Figure 4A). Similarly, subjective muscle soreness, as assessed using a 100-mm visual analogue scale, showed a significant increase at 0- and 24-h post-exercise (*p* < 0.05) across all groups, with no significant intergroup differences observed at any time point (Figure 4B).

## 4 Discussion

This study examined the effects of KT applied either before (KT-pre) or immediately after (KT-post) eccentric exercise on knee extensor performance and markers of muscle damage in healthy young males. Overall, our repeated-measures, counterbalanced design demonstrated that an eccentric exercise protocol produced significant acute reductions in muscle performance—specifically, decreases in MVIC and the RFD, as well as significant increases in subjective muscle soreness and decreases in active ROM at 0- and 24-h post-exercise. Although the KT-pre condition exhibited a relatively smaller decline in performance immediately following exercise, these improvements were not sustained at later time points, suggesting that the protective effect of pre-exercise taping is transient.

In terms of knee extensor performance, MVIC normalized to body weight and RFD in the 0–200 ms interval both declined significantly at 0- and 24-h post-exercise across all groups. These findings corroborate previous reports that eccentric exercise results in immediate impairments in muscle force and explosive capacity (Chen et al., 2019). Notably, the KT-pre group exhibited a less pronounced decline in MVIC and RFD immediately post-exercise, which may indicate that applying KT prior to exercise can provide some mechanical support or enhance proprioceptive feedback during the eccentric load. For example, Li et al. (2024) observed that pre-application of KT mitigated acute force loss in the quadriceps, suggesting that enhanced sensory input might help maintain neuromuscular activation (Li et al., 2024). However, the absence of sustained differences among the groups at 24 and 48 h indicates that the initial benefits of KT-pre do not extend into the longer recovery phase.

Muscle damage indicators further confirm the impact of the eccentric protocol. Active ROM, as measured via goniometry, decreased significantly at 0- and 24-h, and subjective muscle soreness on a 100-mm visual analogue scale increased significantly at these time points across all groups. These changes are consistent with recent studies that have documented significant transient impairments in joint mobility and increases in pain perception following eccentric exercise (Ghai et al., 2024). Despite the robust within-group changes from baseline, no significant intergroup differences were detected, suggesting that neither KT-pre nor KT-post substantially attenuates the overall muscle damage associated with eccentric exercise. This observation aligns with findings by Ozmen et al. (2016), who reported minimal differences in clinical markers of muscle damage between taped and non-taped conditions in the lower extremities (Ozmen et al., 2016).

The transient protective effect observed with KT-pre may be attributed to its potential to enhance proprioceptive feedback and provide mechanical support during eccentric contractions (Ataş et al., 2024). Additionally, patellar stability may play a crucial role in modulating the mechanical support offered by KT, particularly during high-load or repetitive knee extension activities. Recent studies have shown that improper alignment or instability of the patella can diminish the effectiveness of KT and even exacerbate joint stress, especially in individuals with prior knee injuries or altered patellofemoral mechanics (Biz et al., 2024; Biz et al., 2021). Ensuring appropriate tape placement and individual anatomical considerations could therefore be essential in maximizing KT efficacy. However, the lack of sustained differences between the taping conditions and the control indicates that the benefits of KT may be limited to the immediate post-exercise phase. Our results are consistent with previous studies reporting mixed outcomes regarding the efficacy of KT in mitigating exercise-induced muscle damage (Li et al., 2024). Several limitations should be considered. The sample size was relatively small and limited to healthy young males, which may affect the generalizability of our findings. Additionally, the follow-up period of 48 h may not fully capture the longer-term recovery processes. Future research should include larger, more diverse populations and potentially extend the observation period to determine if longer-term benefits of KT exist. Moreover, investigating the underlying mechanisms—such as alterations in peripheral circulation and proprioceptive acuity—may elucidate how KT influences neuromuscular recovery.

## 5 Conclusion

In this study, KT-pre offered a brief protective effect against eccentric exercise-induced declines in knee extensor performance. However, neither KT-pre nor KT-post led to sustained improvements in muscle damage indicators or recovery measures at later time points. These findings suggest that while KT may provide short-term benefits immediately after eccentric exercise, it does not substantially enhance long-term recovery. Future research with larger samples and extended follow-up is needed to clarify KT’s mechanisms and its practical utility in both athletic and clinical contexts.

## Data Availability

The original contributions presented in the study are included in the article/supplementary material, further inquiries can be directed to the corresponding author.
